# Changes in sensitivity and hedonic rating to transcutaneous electrical nerve stimulation following COVID-19

**DOI:** 10.1038/s41598-024-51596-5

**Published:** 2024-01-12

**Authors:** Grzegorz Błażejewski, Joanna Witkoś, Magdalena Hartman-Petrycka

**Affiliations:** 1 Faculty of Medicine and Health Science, Andrzej Frycz Modrzewski Krakow University, Kraków, Poland; 2 Department of Basic Biomedical Science, Faculty of Pharmaceutical Sciences in Sosnowiec, Medical University of Silesia, Katowice, Poland

**Keywords:** Health care, Health occupations

## Abstract

COVID-19 affects not only the respiratory system but also other biological systems such as the nervous system. Usually, these changes are reported based on the patient's subjective description. The aim of our study, therefore, was to objectively determine the effect that the SARS-CoV-2 virus and COVID-19 disease has on sensory threshold and the hedonic and subjective perception of an electrical stimulus. The sensory threshold was tested on the inner forearm by applying non-invasive transcutaneous electrical nerve stimulation (TENS) with 100 Hz and 100 µs parameters and a biphasic current waveform. The study involved 211 participants, aged 22–79 years, with a mean age of 56.9 ± 12.1 years. There were 131 subjects in the COVID group, while the NON-COVID group, the control group, was matched to the COVID group in terms of gender, age, body mass index and presence of chronic diseases. The research was carried out in 2022. Sensory sensitivity was highest in the group that had suffered with COVID-19. The median sensory sensitivity was 11 mA in the COVID group and 14 mA (*p* < 0.001) in the NON-COVID group, however, the current sensitivity threshold decreased over time (R = 0.52, *p* < 0.001). Post COVID-19, the electrical stimulus was more often perceived as unpleasant: COVID versus NON-COVID (23% vs. 3%, *p* < 0.001) and as a different sensation to tingling (27% vs. 2%, *p* < 0.001). Post-COVID-19 patients have a lower sensory threshold, the electrical stimulus is more often described as unpleasant and in subjective feelings it is more often described as pinching. The differences between COVID and NON-COVID decrease with time since the onset of COVID symptoms.

## Introduction

COVID-19 is a respiratory infection caused by the SARS-CoV-2 virus, usually manifested by a dry cough, fever and dyspnoea. This virus can also cause acute respiratory distress syndrome (ARDS), which caused an alarming number of deaths which has been increasing with each wave of successive mutations (Delta, Omicron)^1–3^. In addition to this, COVID-19 may also affect other systems in the human body, including the nervous system and the cardiovascular system, contributing to multiple organ failure and eventual death^4^.

There are increasing reports in the literature of neurological changes caused by the SARS-CoV-2 virus, which can infect the central nervous system (CNS) via both transneuronal and haematopoietic routes^5–8^. The most commonly described neurological symptoms are headache, ataxia and seizures associated with the entry of the SARS-CoV-2 virus into the CNS and loss of sense of smell (anosmia) and taste (ageusia) associated with the peripheral nervous system (PNS) infection^9–12^. The symptoms described above can occur before, during or even after the patient’s respiratory system is infected with SARS-CoV-2^13^. Furthermore, it has been found that patients with a severe COVID-19 infection have more neurological abnormalities than those with a mild infection^7^. Anosmia has a direct association with the PNS and, when present in patients with COVID-19, may explain and give a clue to the likely mechanism of the SARS-CoV-2 virus entry into the brain via the olfactory nerves. Initial neuroinvasive hypotheses included the ability of SARS-CoV-2 to directly access the CNS by invading the olfactory bulb^11^. The piriform cortex may be the virus' main route into the brain^14,15^. Currently, it is thought that the spread of the virus from the environment to the CNS is possible via the sensory afferents which supply the trigeminal nerve, the lingual-pharyngeal nerve, the vagus nerve and the dorsal root ganglia (DRG)^16^. Another hypothesis for the penetration of the SARS-CoV-2 virus into the CNS is that it is possible through secondary systemic mechanisms such as inflammatory cytokines, hypoxemia and oxidative stress associated with acute respiratory distress syndrome^17^.

Peripheral neuropathies appeared as a specific COVID-19 symptom in 59% patients^18,19^. The SARS-CoV-2 virus enters cells via the angiotensin converting enzyme 2 (ACE2) receptor with which the virus spike protein binds^20^. Studies suggest that neurons are not highly infected with SARS-CoV-2 due to the lack of ACE2 expression, and that neuropathies may be caused by an inflammatory response affecting sensory neurons in a cell-autonomous manner^19,21,22^. Studies by Shiers et al.^20^ have shown that about a quarter of human DRG neurons express ACE2 mRNA, and that ACE2 protein is also found in human DRG. Most of these neurons are nociceptors, which are thought to form free nerve endings in the skin or other organs, providing the entry point for the virus into the PNS.

There are no studies in the literature that identify the sensory sensitivity of COVID-19 survivors, which is directly related to any changes in the PNS. The two main aims of the research, therefore, were to provide an objective assessment, using TENS, of the effect of COVID-19 on the sensory threshold, understood in this study as the minimum level of a consciously perceived electrical stimulus and to demonstrate the changes that may occur in the cutaneous sensory area and thus the effect of the SARS-CoV-2 virus on the PNS in COVID-19 patients. The subjective and hedonic interpretation of the sensations produced by the applied electrical stimulus in the subject was also assessed. An additional aim was to determine whether the time elapsed between the start of the disease, that is the time from the onset of the first acute symptoms of COVID-19, to the date of the objective measurement of the sensory threshold, affected the sensation of the applied electrical stimulus.

## Methods

### Key elements of the study design

The study included a medical history taken from the people who had been referred for TENS physiotherapy for pain.

The exclusion criteria for the study were absolute contraindications to physiotherapy and electrotherapy procedures, i.e. sensory disturbances, discontinuity of the skin at the treatment site, heart disease, including the presence of metal implants (pacemakers), other included contraindications were tumours and conditions after their removal, and, in women, pregnancy. None of the exclusion criteria above applied to any of the patients referred for TENS treatment. Those who had had COVID-19 underwent an extended health interview with questions strictly related to the disease, including questions about the time elapsed since the first acute symptoms of the disease had appeared. On the day of patient qualification for the study, sensory threshold was measured according to the research procedure described in the next section, and information was collected on the hedonic and subjective sensation evoked by TENS.

### The sensitivity measuring procedure

To assess the level of sensory threshold transcutaneous electrical nerve stimulation (TENS) was used. TENS is a non-invasive technique used for pain relief in clinical practice. Electrical current is delivered across the intact surface of the skin to active underlying nerves^23^. In this study, conventional TENS was used: low-intensity, high-frequency 100 Hz and 100 µs parameters and biphasic current waveform. The test was performed on the right upper limb, on the wrist flexor muscle group. The forearm was placed on the table in supination. The electrodes used for the test were 12 cm^2^ in size and were placed in pads soaked in warm water. The intensity of the applied current, expressed in milliamperes (mA), was increased in increments of 0.1 mA until the subject reported a minimal, consciously felt electrical stimulus. When a signal indicated that the subject was feeling the applied electrical stimulus, the current intensity displayed on the apparatus was noted. The patient was then asked to indicate whether the stimulus was pleasant, neutral or unpleasant, and to describe the stimulus by choosing one of the following words: tingling, pricking, scratching, warmth/burning, pinching, numbness and tickling. The research was carried out in 2022.

Use of TENS in the assessment of the sensory threshold is a subjective method, requiring an alert and co-operative patient. The method measures the level at which a subject reports sensing a physical stimulus (detection threshold) applied to the skin and is similar to Quantitative Sensory Testing (QST)^24^ which is a subjective, psychophysical test developed to assess sensory nerve function and measure the level at which a subject reports sensing a physical stimulus (detection threshold) applied to the skin. TENS has been previously used in studies connected with the sensation of electrical stimulation. The studies were conducted on young and healthy subjects in order to assess gender differences in the sensation of external stimulation^25–27^.

Other methods of sensory testing can also be found in literature e.g. the “Gold Standard” for testing cutaneous sensation with calibrated von Frey filaments^28^, which is a tactile method used to perceive surface sensations (tactile sensation) and the Semmes–Weinstein monofilament test to assess the threshold of tactile sensitivity on the skin by using accurate handheld calibrated nylon thread that buckles once it has delivered a force of 10 g^29^. Tuning forks, which are the most commonly clinically used tool to test for vibratory sensation should also be mentioned^30^.

Participants in the study.

**Figure 1:**
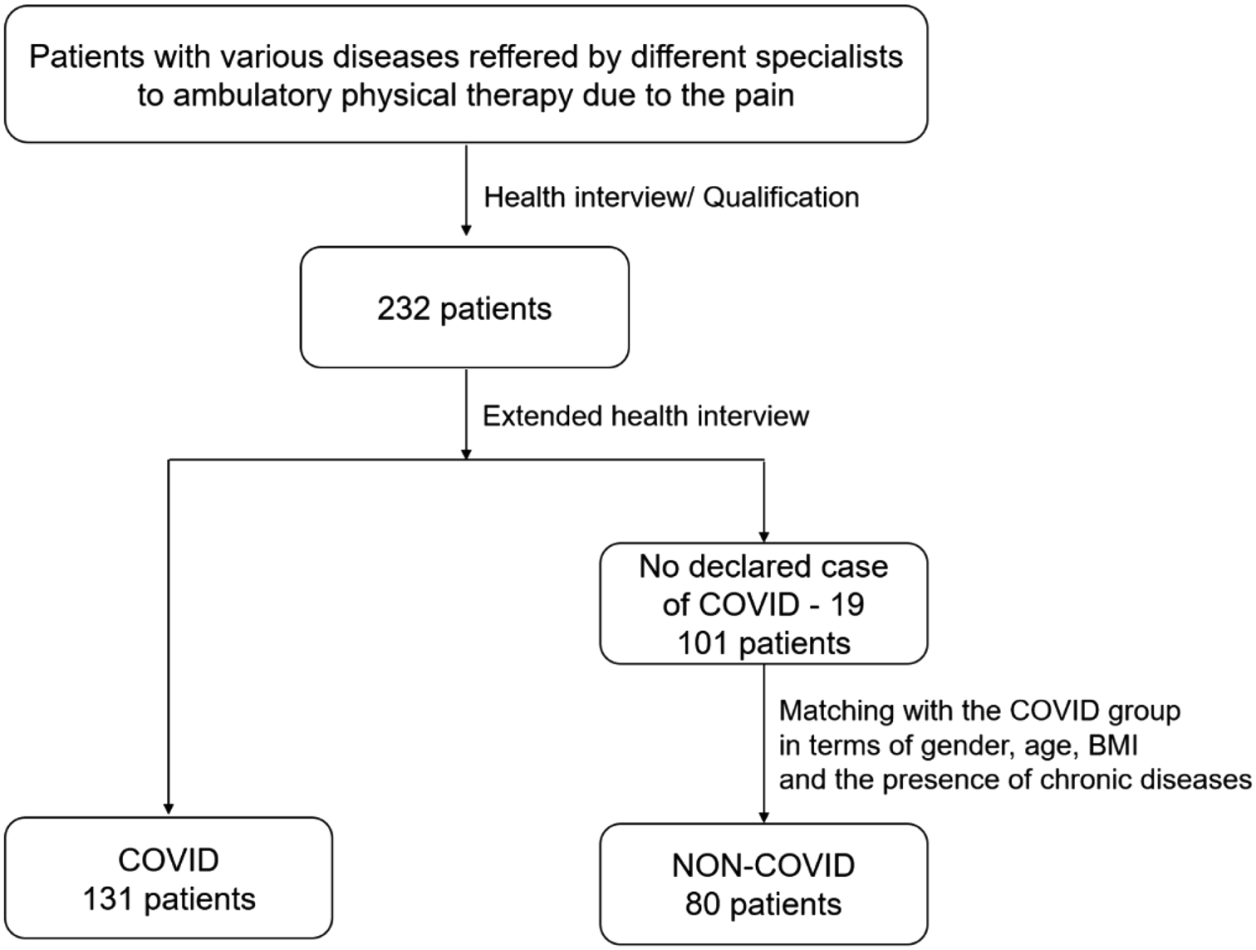
Flowchart of the study patients’ recruitment.

The method used for the inclusion of the study participants in the study groups is shown in Fig. 1.

The final analysis included results from 211 people aged 22 to 79 years, mean ± standard deviation 56.9 ± 12.1 years. There were 110 women and 101 men and body mass index (BMI) was 25.5 ± 2.0 kg/m^2^. They included patients presenting for outpatient physiotherapy for: 67 (31.8%) spinal degeneration, 27 (12.8%) back pain, 27 (12.8%) shoulder injury, 26 (12.3%) multi-joint degeneration, 18 (8.5%) gonarthrosis, 15 (7.1%) ankle sprain, 10 (4.7%) knee sprain, 10 (4.7%) other soft tissue disease, 7 (3.3%) coxarthrosis, 3 (1.4%) internal knee injury, 1 (0.5%) acquired musculoskeletal deformity.

From this group of people, 131 declared that they had had COVID-19 and formed the COVID group, while the NON-COVID group of 80 people consisted of people who had not had COVID-19, thus forming the control group. Cutaneous sensation may deteriorate for many reasons including: age, contributing morphological changes, decreased numbers of both myelinated afferent fibres and cutaneous receptor end-organs, changes in the mechanics of the skin itself, including dehydration, altered skin elasticity, or poor peripheral blood flow^28^. Therefore the control group was selected to ensure that it had no statistically significant differences from the COVID group in terms of gender, age, height, BMI, presence of chronic diseases, regularly used medication and types of medication. The characteristics of the COVID and NON-COVID groups are shown in Tables 1 and 2.

**Table 1:** Characteristics of age, height and BMI values in COVID and NON-COVID groups.

	Me	Q1	Q3	Min	Max	Average	SD	*p*
Age [years]								
COVID	61.0	49.0	65.0	22.0	75.0	57.0	11.8	0.993
NON-COVID	60.5	49.0	65.5	25.0	79.0	56.9	12.8	
Height [cm]								
COVID	170.0	167.0	174.0	159.0	182.0	170.2	5.1	0.646
NON-COVID	170.0	165.0	174.5	160.0	182.0	170.0	5.5	
BMI [kg/m^2^]								
COVID	26.4	25.4	27.6	23.0	32.0	26.6	2.0	0.969
NON-COVID	26.5	24.9	28.0	22.7	32.8	26.5	2.1	

*Me* median, *Q1* first quartile, *Q3* third quartile, *Min* minimum, *Max* maximum, *SD* standard deviation, *p *level of statistical significance (Test: Mann–Whitney U).

**Table 2:** Number and percentage of subjects in COVID and NON-COVID groups by sex, BMI categories and chronic diseases, as well as time since COVID-19 onset; *p *level of statistical significance (Test: Chi^2^).

	COVID	NON-COVID	*p*
Sex			
Female	67 (51.1)	43 (53.8)	0.713
Male	64 (48.9)	37 (46.3)	
BMI categories			
Healthy Weight (18.5–24.9)	29 (21.1)	21 (26.3)	0.456
Overweight (25.0–29.9)	92 (70.2)	56 (70.0)	
Obesity (> 30.0)	10 (7.6)	3 (3.8)	
Elapsed time from onset of first acute symptoms of COVID-19			
< 1 month	23 (17.6)	–	–
1–4 months	29 (22.1)		
4–7 months	3 (2.3)		
7–10 months	28 (21.4)		
> 10 months	48 (36.6)		
Chronic diseases			
Hypertension	33 (25.2)	18 (22.5)	0.658
Diabetes	7 (5.3)	3 (3.8)	0.597
Osteoporosis	1 (0.8)	0 (0.0)	0.433
Gout	10 (7.6)	5 (6.3)	0.704
Regularly used medication			
Yes	46 (35.1)	21 (26.3)	0.180
Type of medication			
Polocard	8 (6.1)	6 (7.5)	0.693
Lernidum	2 (1.5)	0 (0)	0.267
Milurit	11 (8.4)	5 (6.3)	0.568
Acard	4 (3.1)	2 (2.5)	0.814
Siofor	1 (0.8)	1 (1.3)	0.723
Nebilet	5 (3.8)	3 (3.8)	0.980
Akineton	4 (3.1)	1 (1.3)	0.403
Adeksa	3 (2.3)	0 (0)	0.173
Metformax	2 (1.5)	0 (0)	0.267
Prestarium	5 (4.6)	0 (0)	0.051
Noriprel	6 (4.6)	6 (7.5)	0.374
Ostenil	1 (0.8)	0 (0)	0.433
Diaprel	2 (0.8)	2 (2.5)	0.301
Telmizek	3 (0.8)	0 (0)	0.433
Formetic	0 (0)	1 (1.3)	0.200

### Statistical analysis

Statistica 13 software (Statsoft, Krakow, Poland) was used for statistical analysis. The Mann–Whitney U test and Spearman's rank correlation coefficient were used due to the lack of normality of the distribution assessed by the Shapiro–Wilk test and the different number of persons in the groups studied. The Chi^2^ test was applied to categorised data. Results were considered statistically significant at *p* < 0.05.

Informed consent.

Informed consent was obtained from all individual participants included in the study. This study was conducted in accordance with the Declaration of Helsinki, and approved by the Bioethical Committee of Andrzej Frycz Modrzewski Krakow University (permission number KBKA/4/O/2022).

## Results

**Figure 2:**
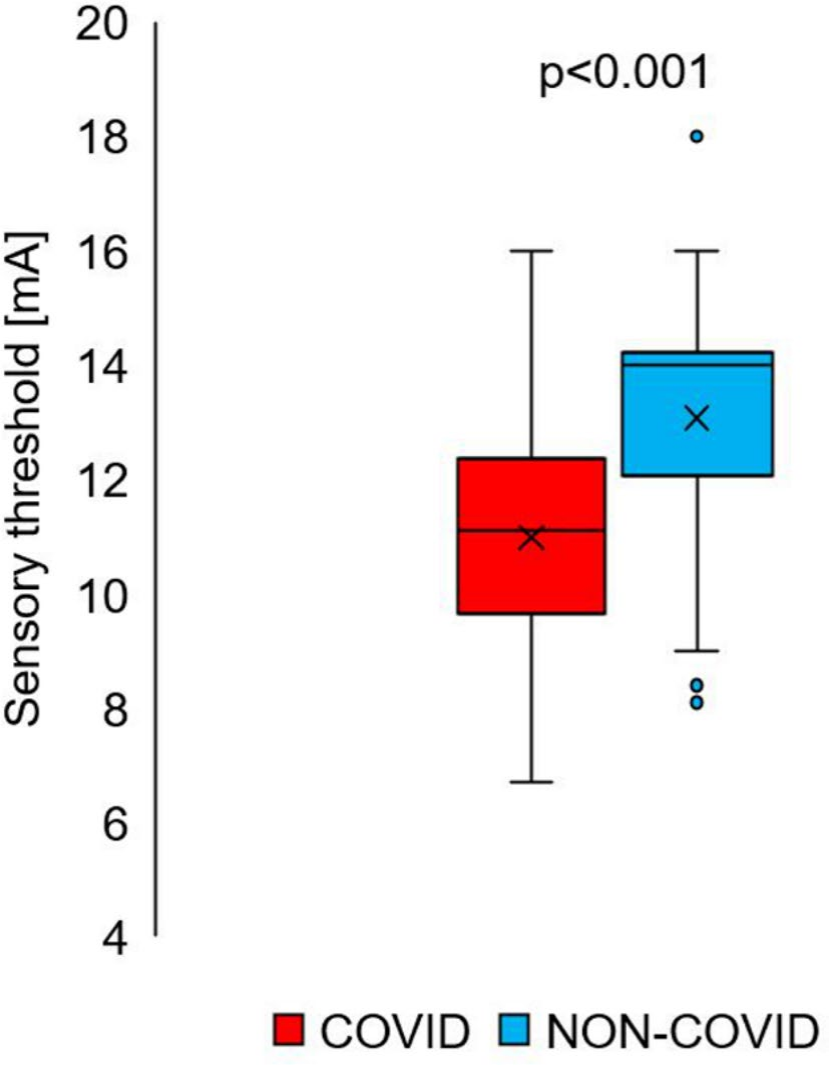
The sensory sensitivity threshold in the group of patients who declared that they had had COVID-19 (COVID), N = 131 and those who declared that they did not have COVID-19 (NON-COVID, N = 80). *x*—average, box—interquartile range, — median (Test: U Mann–Whitney).

**Figure 3:**
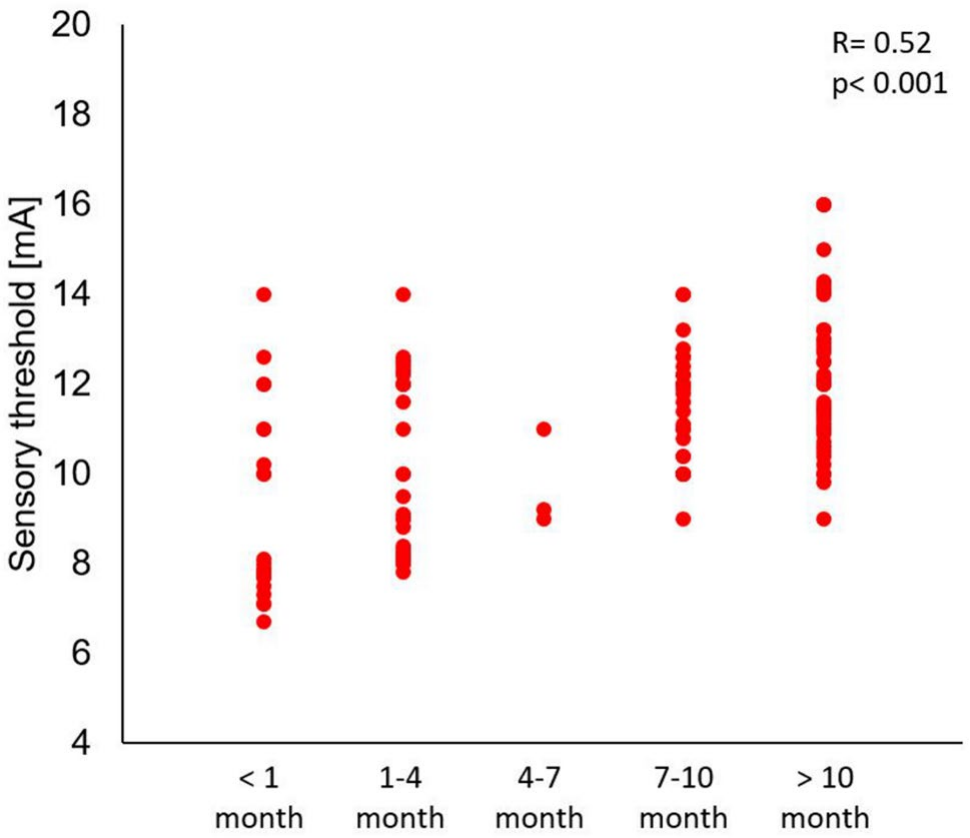
The effect of the time lapse after the first acute symptoms of COVID-19 on the threshold of sensory sensitivity (R Spearman).

A prior COVID-19 infection had a statistically significant impact on the changes in sensory sensitivity (*p* < 0.001) (Fig. 2). There was an increase in skin sensitivity, as assessed by electrical stimulation. The median sensory sensitivity in the COVID group was 11 mA and in the NON-COVID group was 14 mA. The time elapsed from the onset of the first acute symptoms of the disease to the date of measurement had a statistically significant effect on the skin sensitivity threshold value (R = 0.52, *p* < 0.001) (Fig. 3). The lowest values of the sensory sensitivity threshold were observed in subjects whose first acute symptoms of COVID-19 disease occurred one month or less from the day the test was performed, the median sensitivity threshold for that time lapse being 7.9 mA (Table 3). As the time from onset to the present measurement increased, the sensitivity threshold value increased. For patients who declared that they had had their first acute COVID-19 symptoms more than 10 months before the measurement, the median sensitivity threshold took on a value of 12.0, but was still lower than the median current sensitivity threshold for NON-COVID subjects of 14.0.

**Table. 3.** The threshold of sensory sensitivity in the group of people who had suffered with COVID-19 at various times before the measurement and in the group who declared that they had not had the illness (NON-COVID).

Time from onset of first acute symptoms of COVID-19	N	Me	Q1	Q3	Min	Max	Average	SD
< 1 month	23	7.9	7.7	11.0	6.7	14.0	9.1	2.1
1–4 months	29	9.5	8.2	12.0	7.8	14.0	10.1	1.9
4–7 months	3	9.2	9.0	11.0	9.0	11.0	9.7	1.1
7–10 months	28	11.7	10.4	12.3	9.0	14.0	11.5	1.3
> 10 months	48	12.0	11.0	13.1	9.0	16.0	12.2	1.7
NON-COVID	80	14.0	12.1	14.3	8.1	18.0	13.1	2.0

*N* number of subjects, *Me* median, *Q1* first quartile, *Q3* third quartile, *Min* minimum, *Max* maximum, *SD* standard deviation.

**Figure 4:**
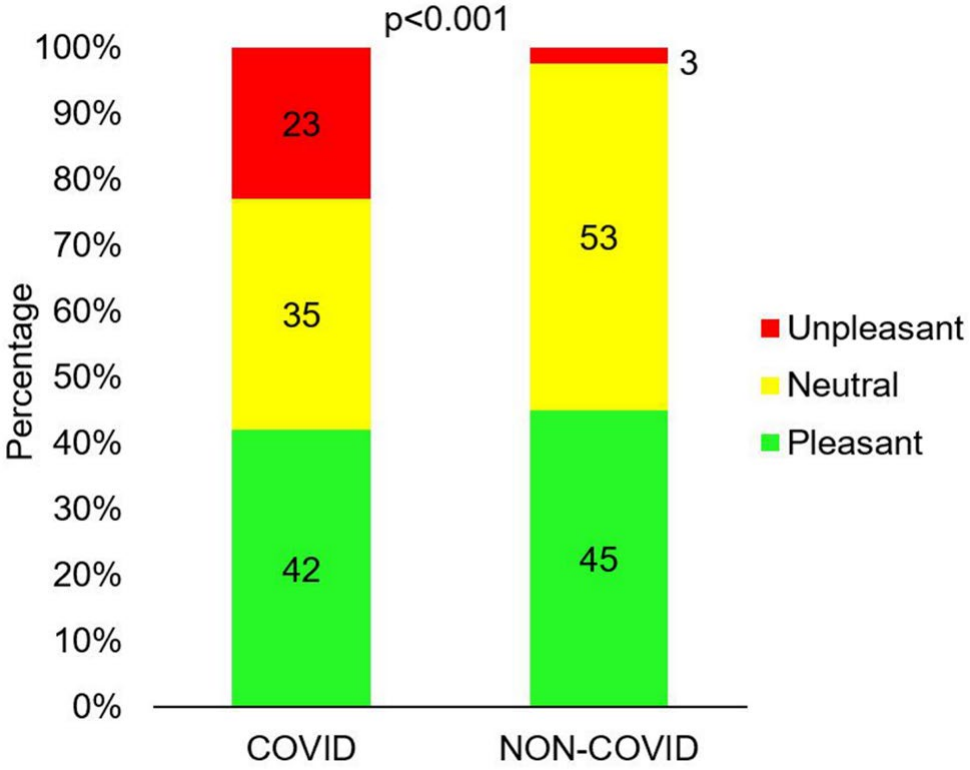
Hedonic rating of the current-induced stimulus for the group of patients who declared that they had had COVID-19 (COVID, N = 131) and those who declared that they had not had COVID-19 (NON-COVID, N = 80) (Test: Chi^2^).

The hedonic evaluation of the stimulus in the group who had had COVID-19 was statistically significantly different from the hedonic evaluation in the NON-COVID group (Fig. 4). Of those who had had COVID-19, the stimulus was perceived as unpleasant by 23%, as neutral by 35%, and as pleasant by 42%, whereas in the group of people who said they had not had COVID-19, only 3% described the stimulus as unpleasant, 53% as neutral and 45% as pleasant. It was observed that the highest proportions of people with an unpleasant perception of the stimulus occurred in the group who had had COVID-19 either up to one month or between one and four months from the day of the measurements. For those with symptoms more than 4 months ago, the predominant response was that the sensation was pleasant (Table 4).

**Table 4:** Hedonic rating of the current-induced stimulus in the group of patients who declared that they had had COVID-19 at various times before the measurement and those who declared that they had not had COVID-19 (NON-COVID).

	< 1 month	1–4 months	4–7 months	7–10 months	> 10 months	NON-COVID
Unpleasant	11 (47.8)	17 (58.6)	0 (0.0)	1 (3.6)	1 (2.1)	2 (2.5)
Neutral	8 (34.8)	8 (27.6)	0 (0.0)	11 (39.3)	19 (39.6)	42 (52.5)
Pleasant	4 (17.4)	4 (13.8)	3 (100.0)	16 (57.1)	28 (58.3)	36 (45.0)

The percentage of individuals is in brackets next to the number of individuals.

**Figure 5:**
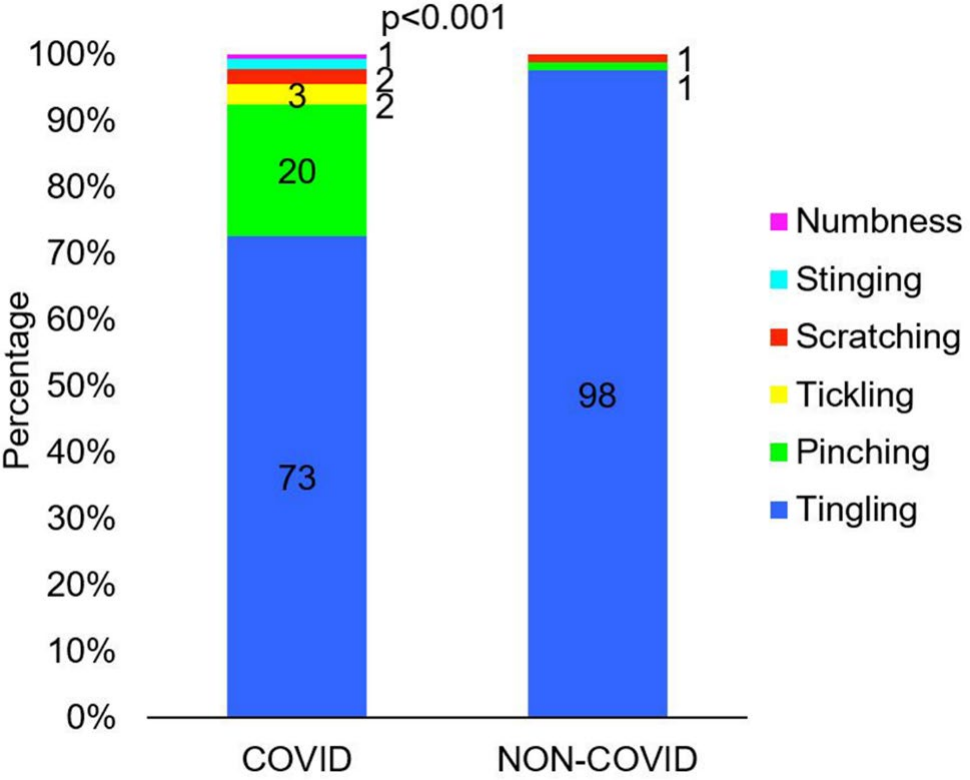
Subjective description of the sensory sensitivity caused by the electrical current in the group of patients who declared that they had had COVID-19 (COVID, N = 131) and those who declared that they had not had COVID-19 (NON-COVID, N = 80) (Test: Chi^2^).

The subjective description of the stimulus felt by COVID-19 survivors differed significantly from the subjective stimulus felt by NON-COVID subjects (*p* < 0.001) (Fig. 5). In the group who had had COVID-19, 73% described the stimulus as tingling, 20% as pinching, with the other terms being used sporadically. In the group of people who declared that they had not had COVID-19 almost all described the sensation as tingling 98%. Taking into account the time lapse between the onset of the first acute symptoms of COVID-19 and the day on which the sensory threshold was measured, those who had been ill either up to one month or between one and four months differed most from the NON-COVID subjects in their subjective perception of the stimulus (Table 5).

**Table 5:** Subjective description of the sensory sensitivity caused by the electrical current in the group of patients who declared that they had had COVID-19 at various times before the measurements were taken and those who declared that they had not had COVID-19 (NON-COVID).

	< 1 month	1–4 months	4–7 months	7–10 months	> 10 months	NON-COVID
Tingling	11 (47.8)	8 (27.6)	3 (100.0)	27 (96.4)	46 (95.8)	78 (97.5)
Pinching	10 (43.5)	15 (51.7)	0 (0.0)	0 (0.0)	1 (2.1)	1 (1.3)
Other	2 (8.7)	6 (20.7)	0 (0.0)	1 (3.6)	1 (2.1)	1 (1.3)

The percentage of people is given in brackets next to the number of people.

## Discussion

This study showed that the longer the time lapse between the first acute symptoms of COVID-19 and the present study of sensitivity to an electrical stimulus, the higher the value of the current required to reach the sensory threshold, indicating a higher sensory threshold and reduced sensory sensitivity. It is not possible to compare the results of our own study with reports from the literature on sensory perception after COVID-19, as this type of study does not appear in the currently available databases of scientific publications. Due to the direct link of anosmia and ageusia with the PNS, it is worth paying special attention to these symptoms^31–34^ which tend to occur in the early stages of the disease, as they may, therefore, be useful diagnostic markers^35^. A comparison was made with data from other disorders of the PNS, i.e. anosmia and ageusia^14,36,37^. A similar time relationship was found regarding the return to normal for the sensory disorders, both taste and olfactory, accompanying COVID-19^14–21^. For example, a study by Qiu et al.^36^ observed that 10% of COVID-19 patients had olfactory or gustatory symptoms only, while 19% reported olfactory and/or gustatory disorders before any other COVID-19 symptom. The range of follow-up time was 3 weeks, during which time 43% of patients experienced improvement in olfactory or taste dysfunction^19^, a similar time to our study where the shorter the time lapse from the onset of the first acute symptoms of COVID-19 the more pronounced were the differences in the perception and evaluation of the stimulus administered with TENS stimulation.

The electrical stimulus which accompanied the TENS stimulation and which was used in the research had a short impulse time expressed in microseconds and a high frequency. This was deliberately designed so that the sensation that accompanied its flow through the skin was a delicate, pleasant tingling sensation. Our own research showed that almost all people who had not had COVID-19 interpreted their sensations in exactly this way. For them, the stimulus was neutral or pleasant. However, it should be noted that in the group of people who had had COVID-19, there were people who also reported other sensations accompanying TENS stimulation, such as: pinching, scratching and stinging. There were also people who reported that they found that the applied stimulus was unpleasant. This hedonic evaluation of the TENS stimulus on the skin indicates that the skin sensitivity of COVID-19 survivors was higher than those who had not had COVID-19, which may support the hypothesis that COVID-19 and the SARS-CoV-2 virus have an effect on the increased sensitivity of survivors.

There is also a lack of clear evidence in the literature on the impact of SARS-CoV-2 virus and, in turn, COVID-19 on the PNS. In a study by Flamier et al.^18^ which aimed to investigate the effects of SARS-CoV-2 on the PNS and try to elucidate the neuropathies associated with COVID-19, human sensory neurons were generated from induced pluripotent stem cells and sequentially infected with SARS-CoV-2 strain WA1/2020 and the Delta and Omicron variants. It was shown that 20% of human sensory neurons were infected with SARS-CoV-2, with the Omicron variant having the lowest infection rate. The study also showed that although SARS-CoV-2 infects human sensory neurons, it does not actively replicate to shed progeny virions.

The first researchers to describe cutaneous hypersensitivity as a clinical symptom of COVID-19 were Krajewski et al.^38,39^, who, two months after the onset of the pandemic, collected data from nine patients, with an average age of 47.7 years who reported an increased sensitivity to stimulation**.** Most of the patients studied also showed the typical general symptoms of COVID-19, such as a dry cough and fever. Cutaneous hyperaesthesia most often appeared 2–3 days after the onset of general disease symptoms, but in one patient it was the first symptom of the disease. Another patient reported increased skin sensitivity 5 days after resolution of general COVID-19 symptoms. The duration of the skin hypersensitivity varied considerably between patients, ranging from one day to six months. However, Harsh et al.^40^ described the case of a 69-year-old female patient with a moderate COVID-19 infection in whom any form of touch exacerbated the phenomenon of significant cutaneous hypersensitivity, particularly in the abdomen and lower extremities. The hypersensitivity resolved spontaneously after eight days. Abrams et al.^41^, retrospectively. examined sensory symptoms in thirteen patients with painful paraesthesia and numbness that developed during, or after, a SARS-CoV-2 infection. Six of the thirteen patients had a definitive diagnosis of small fibre neuropathy, including two cases with dysautonomia on autonomic testing. Nerve conduction studies showed no evidence of large fibre polyneuropathy. Studart-Neto et al.^42^ found that three (6.7%) of 45 COVID-19 patients with severe respiratory disease developed peripheral neuropathy. Mehan et al.^43^ assessed patients with myositis after COVID-19, including lower extremity paraesthesia and back pain. After magnetic resonance imaging revealed oedema and enhancement of the paraspinal muscles, it was hypothesised, that myositis may be relatively common in COVID-19 patients and that paraesthesia in these patients may be due to this myositis. Andalib et al.^44^ reviewed the literature on PNS symptoms associated with COVID-19. It was concluded that the reason for these PNS symptoms was because of dysregulation of the systemic immune response by the COVID-19 virus. It has also been proposed that, after the acute phase of the infection has passed, patients with COVID-19 often go on to develop systemic excessive inflammation with macrophage activation, also known as secondary hemophagocytic lymphohistiocytosis.

### Limitations

The methodology used in this study makes it possible to conclude unequivocally that a history of COVID-19 influences the sensations associated with the application of an electrical stimulus using TENS. However, the main limitation of the present study was the fact that only a single measurement of current sensation was carried out on each patient, which makes it impossible to precisely determine changes in the perception of the stimulus over time, as the passage of time is influenced by various individual factors. Unfortunately, for practical reasons, it was not possible to perform multiple measurements on the same person, which would have increased the reliability of the conclusions. Furthermore, the time since COVID-19 was measured using intervals. An additional limitation of this study was that the SARS-CoV-2 virus subvariant could not be determined, and it is known^45^ that disease symptoms are subject to modification in the case of different subvariants. Moreover, based only on an interview with the participant, the final classification into COVID and NON-COVID may have been subject to error, since the group of people who declared that they had not had COVID-19 may have included people who had had the disease asymptomatically without being aware that they had it. The conclusions presented below must be read in the light of these limitations. There are no studies in literature on sensory changes in post-COVID-19 patients, so there is a need for further research and comparison of results obtained by researchers and clinicians on the occurrence of these sensory changes (cutaneous hypersensitivity) in patients who have or have had COVID-19. Further evidence is needed to determine the prevalence of this phenomenon in COVID-19 patients, how long it takes to resolve and to clarify its pathogenesis.

## Conclusions

Having COVID-19 causes a reduction in the threshold of sensory sensitivity, a more unpleasant perception of the electrical stimulus and a change in the subjective sensations induced by the current from tingling to pinching and/or other unpleasant sensations. The shorter the time lapse from the onset of the first acute symptoms of COVID-19 in the group of people who declared that they had suffered from the disease, the more pronounced were the differences in the perception and evaluation of the stimulus administered with TENS stimulation compared to the group of people who declared that they had not suffered from COVID-19. Thus, the study observed a hypersensitivity to sensory stimuli that diminished with time from the onset of the first acute symptoms of the disease.

## Data Availability

The datasets used and/or analysed during the current study available from the corresponding author on reasonable request.
